# A New Cross-By-Pass-Torus Architecture Based on CBP-Mesh and Torus Interconnection for On-Chip Communication

**DOI:** 10.1371/journal.pone.0167590

**Published:** 2016-12-01

**Authors:** Usman Ali Gulzari, Muhammad Sajid, Sheraz Anjum, Shahrukh Agha, Frank Sill Torres

**Affiliations:** 1 Department of Electrical Engineering, COMSATS Institute of Information Technology, Islamabad, Pakistan; 2 Department of Computer Science, COMSATS Institute of Information Technology, Wah Cantt, Pakistan; 3 Department of Electronic Engineering, University of Minas Gerais, Minas Gerais, Brazil; West Virginia University, UNITED STATES

## Abstract

**A** Mesh topology is one of the most promising architecture due to its regular and simple structure for on-chip communication. Performance of mesh topology degraded greatly by increasing the network size due to small bisection width and large network diameter. In order to overcome this limitation, many researchers presented modified Mesh design by adding some extra links to improve its performance in terms of network latency and power consumption. The Cross-By-Pass-Mesh was presented by us as an improved version of Mesh topology by intelligent addition of extra links. This paper presents an efficient topology named Cross-By-Pass-Torus for further increase in the performance of the Cross-By-Pass-Mesh topology. The proposed design merges the best features of the Cross-By-Pass-Mesh and Torus, to reduce the network diameter, minimize the average number of hops between nodes, increase the bisection width and to enhance the overall performance of the network. In this paper, the architectural design of the topology is presented and analyzed against similar kind of 2D topologies in terms of average latency, throughput and power consumption. In order to certify the actual behavior of proposed topology, the synthetic traffic trace and five different real embedded application workloads are applied to the proposed as well as other competitor network topologies. The simulation results indicate that Cross-By-Pass-Torus is an efficient candidate among its predecessor’s and competitor topologies due to its less average latency and increased throughput at a slight cost in network power and energy for on-chip communication.

## Introduction

The growing complexity of System-on-Chip (SoC) designs, characterized by an increasing number of Processing Elements (PEs), requires intelligent solutions for on chip communication. In alignment with this challenge, Networks-on-Chip (NoC) is emerging as a new and promising paradigm that targets an efficient communication between PEs [1]. NoC-based systems appear as an enhanced solution, as an evolution of flexibility, multitasking parallel computing, data capacity and scalability for future on-chip communications [2]. It uses packet switching and routing technology to reduce power consumption, to improve reusability, reliability and performance [3–4].

Customarily, topology is an important factor in a design which affects the overall performance of the NoC [5–6]. The efficient design of topology plays a role as a backbone to the complete NoC structure [7]. Topology design not only reflects the connection of each module distribution, it is also responsible for data transmission on chip [8]. Therefore, topology design plays an essential role in the performance of on chip communication network [9]. The performance of latency, throughput and other parameters are mainly dependent on the hop counts by a packet which traverses from source to its destination in the designed network. A topology has high impacts of power, energy, latency and throughput, but also on the routing and mapping strategy [10–13]. Principal issues to be addressed in NoC are reduction of power consumption and energy utilization at low penalties in performance, latency and throughput [14]. Further issues are network scalability and design complexity of routing elements [15–16].

The most promising and widely applied NoC topology is the so-called Mesh, which profits from a regular and simple structure [17]. However, Mesh networks suffer under poor scalability for large amount of PEs due to the great number of multi-hop links needed to provide complete reachability [18]. In order to overcome this limitation, many researchers presented modified Mesh design by adding some extra links to reduce the network diameter and increase the bisection width that in turn improves the overall performance of the network. Some alternative solutions are Meshes with hierarchical topologies like D-Mesh and D-Torus [19], which reduce the average hop count in the NoC. However, proposed structures lead to increased router complexity as well as higher costs in terms of power and energy consumption [20–21].

This paper presents an efficient and scalable Cross-By-Pass-Torus (CBP-Torus) architecture for on-Chip communication, which is the upgraded version of C^2^-Torus and CBP-Mesh [22–23]. The architecture of the proposed network topology is based on the basic Mesh topology, with Cross-By-Pass-Links (CBP-Links) similar to the Cross-By-Pass-Mesh (CBP-Mesh) topology [23]. The Torus-Links (T-Links) are added in the terminal nodes of the network, which reduces the network diameter and average hop count between the nodes in the proposed network. The additions of CBP- and T-Links provide reduced paths for packets between source to destination nodes and increase the performance of the network w.r.t its predecessor’s and competitors topologies. In order to get an extensive analysis of the proposed CBP-Torus, the synthetic uniform random traffic and five different embedded application workloads were applied. The proposed topology design is compared with some of its predecessor’s such as Mesh, Torus, C^2^-Torus and CBP-Mesh topologies as well its competitors D-Torus [18–23]. The simulation results indicate that Cross-By-Pass-Torus is an efficient candidate among its predecessor’s and competitor topologies due to its less average latency and increased throughput at the cost of a slight increase in network power and energy for on-chip communication.

This study is structured as follows. Section-II highlights, background work. Section-III describes the architecture of the CBP-Torus. Section-IV address CBP-Torus features. Section-V is based on performance result of topologies versus energy and power overhead cost. Lastly, section-VI concludes this work.

## Background

**Fig 1:**
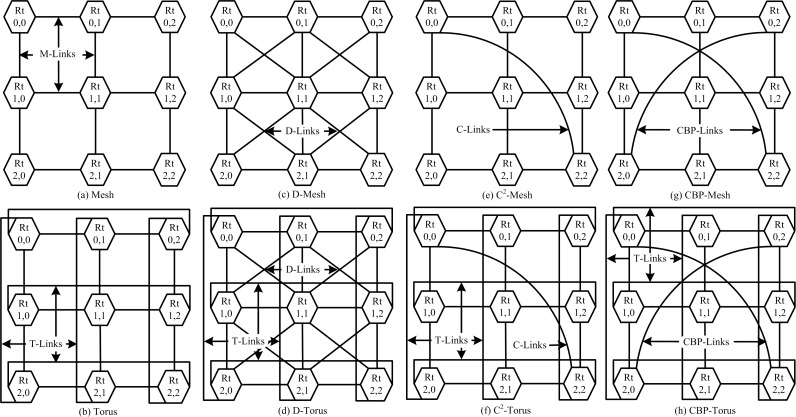
**(a-h):** Eight different types of 3x3 topologies with routers and interconnected links.

The multi-core system and on-chip communication widely uses direct interconnection networks. Mesh is best example of direct interconnection network [19] depicted in Fig 1(A). Many general purpose applications use Mesh topology due to its simple and regular network design [22]. By engaging a large number of PEs on Mesh network, its performance is degraded due to increase in network diameter size having small bisection width [19]. Many authors presented different architectures by modifying Mesh design, adding links on network for improving the performance. To improve the Mesh performance, Torus topology added T-Links connected to all terminal node pairs to reduce the network diameter shown in Fig 1(B) [19]. Diagonal Mesh (D-Mesh) and Diagonal Torus (D-Torus), shown in Fig 1C and 1D) introduced additional diagonal links to reduce network diameter and to reduce the network latency [20–21]. D-Mesh can be constructed by adding D-Links on simple Mesh. D-Mesh comprises of nine degree inner nodes, which reduces the average hop counts of the network at the cost of network power [18]. D-Torus merged links of D-, T- and M-Links on a one network, Fig 1(D). D-Torus achieved high performance from topologies like Mesh, X-Mesh, D-Mesh and SD-Torus as a comparison in [18]. Consequently, the cost of D-Torus network is drastically increased in terms of area and power consumption [18, 21]. Hence, high degree routers are required to implement D-Mesh and D-Torus network topologies leading to increasing the cost of power consumption [18].

Center-Connected Mesh (C^2^-Mesh) and Center-Connected Torus (C^2^-Torus) networks shown in Fig 1E and 1F) are based on simplicity and cost effectiveness features [19, 22]. Additional four Cross-links (C-Links) on 5×5 Mesh network centrally interconnect nodes in C^2^-Mesh whereas in C^2^-Torus topology all terminal node pairs are also connected to the network. The C^2^-Mesh and C^2^-Torus networks are simple and have low cost; however, their performances are less efficient in comparison to D-Mesh and D-Torus topologies [23]. Therefore, efficient, high performance and low cost network architecture are required to account for ever increasing number of PEs. A CBP-Mesh [23] is upgraded to C^2^-Mesh with improved performance as compared to its predecessors, which is depicted in Fig 1(G). CBP-Links in CBP-Mesh are more effective to reduce the average latency and improves cost effectiveness as compared to D-Mesh and D-Torus [23]. Fig 1A–1H) depicts 3×3 node’s network regarding Mesh, Torus, D-Mesh, D-Torus, C^2^-Mesh, C^2^-Torus, CPB-Mesh, and proposed CBP-Torus topologies where the hexagonal box router (Rt) and interconnected links represent these networks.

## CBP-Torus Architecture

To increase the performance of Mesh network, the worst case scenario of hop count for Mesh should be addressed. The worst cases in hop count of Mesh topology include the opposite corner nodes (Rt_0,0_ ↔ Rt_2,2_ and Rt_0,2_ ↔ Rt_2,0_ in Fig 1A) which are four in a 3×3 network. By using the T-links in Torus network, it covers this distance in two hops by intersecting corner nodes. A D-Mesh and D-Torus topologies also take two hops to traverse a packet to its opposite corner node using D-Links. In case of a 3×3 Mesh network, C^2^-Mesh uses one extra C-Link that reduces the hop count between two opposite corner nodes (Rt_0,0_ ↔ Rt_2,2_ in Fig 1E). However, the communication between the second opposite corner node is not affected (Rt_0,2_ ↔ Rt_2,0_ in Fig 1E). C^2^-Torus connects the terminal with T-Links to reduce the distance between the opposite terminal nodes for an increase in the performance of the network. In CBP-Mesh design the two CBP-Links are added to a Mesh network, placed between both pairs of opposite corner nodes (Rt_0,0_ ↔ Rt_2,2_ and Rt_0,2_ ↔ Rt_2,0_ in Fig 1G) and minimizes two to one hop against the Torus, D-Mesh, D-Torus and connecting other side of nodes Rt_0,2_ ↔ Rt_2,0_ from C^2^-Mesh, C^2^-Torus networks. Consequently, all four corner nodes are interlinked in the 3×3 CBP-Mesh by over-passing the central node (Rt_1,1_ in Fig 1G). CBP-Links also reduces the distance between Rt_0,0_ ↔ Rt_1,2_, Rt_2,1_ by hopping Rt_2,2_ to two hops. Similarly, all the corner nodes can access the other side of both middle nodes Rt_0,1_ Rt_1,0_ Rt_1,2_ Rt_2,1_ and vice-versa in one hop that leads to higher performance in the network [23].

The proposed network is the Torus version of CBP-Mesh. T-Links added to CBP-Torus connects the terminal nodes as shows 3×3 network in Fig 1(H). The addition of T-Links reduces the network diameter from the terminal sides of the proposed network. T-Links also provide multipath along with the CBP-Link and M-Links in the proposed network and helps to accommodate more adaptive and dynamic routing algorithms in the network.

### CBP-Torus Design

The placement of links is a fundamental design step in CBP-Torus network. The proposed CBP-Torus comprises of features attributed to three types of links, including Mesh-Links (M-Links), T-Links) and CBP-Links.

**Fig 2:**
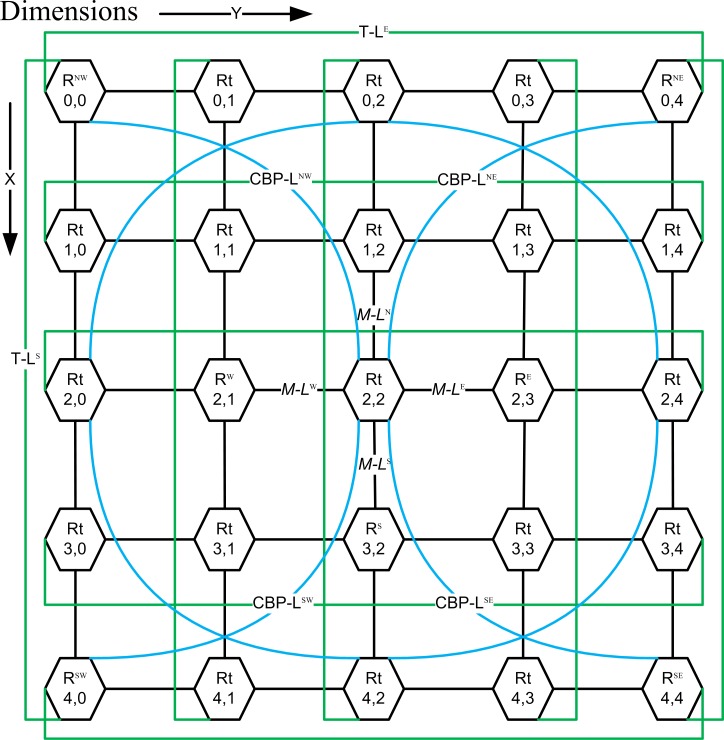
The 5×5 CBP-Torus network depicting Rt routers with connecting links

**Fig 3:**
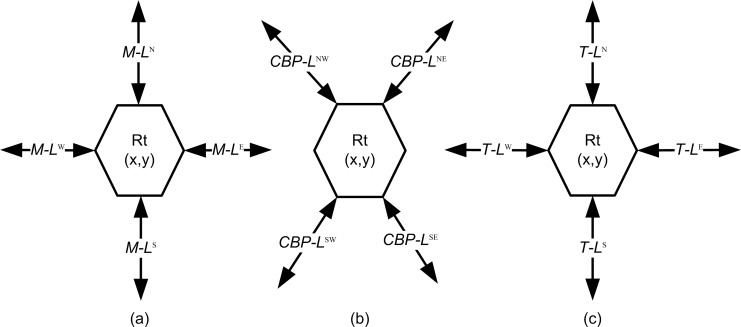
The CBP-Torus router (Rt) with router links for interlinking the connection with neighbors router nodes. a) Rt with M-Links, b) Rt with CBP-Links, c) Rt with T-Links.

The blue and green lines in Fig 2, represent CBP-Links and T-Links for proposed CBP-Torus embedded on Mesh topology. The proposed CBP-Torus can be defined as an embedded CBP- and Torus links on a two-dimensional Mesh network with coordinates of m × n where m and n are the number of rows and columns respectively. A node (*N*) in CBP-Torus consists of two coordinates x and y represented as *N*_*x*,*y*_ *= {(x*, *y) | 0 ≤ x ≤ m-1*, *0 ≤ y ≤ n-1}*. In CBP-Torus, each node has its own router Rt_x,y_ and interlinks neighbors with each other by a horizontal and vertical M-links in the network. Router possible neighbors are on the North (R_N_), South (R_S_), East (R_E_) and West (R_W_) and linked by using M-Links (M-L^N^, M-L^S^, M-L^E^ and M-L^W^) represented by Rtx,y in Fig 3(A). The proposed CBP-Torus network links design of the CBP- and T-Links and can be defined as follows;

**Definition 1.** *For CBP-Links router Rt*_*x*,*y*_ *if both x and y coordinates are even numbers*, *then CBP-Links are connected to router ports with coordinates (x+2*, *y+2)*, *(x-2*, *y+2)*, *(x+2*, *y-2) and (x-2*, *y-2) by CBP-L*^*SE*^, *CBP-L*^*SW*^, *CBP-L*^*NE*^, *and CBP-L*^*NW*^ *links*.

**Definition 2.** *For Torus-Links router Rt*_*x*,*y*_ *if x or y coordinates are terminal nodes of the network then T-Links are added to the network*. *The terminals Rt*_*x*,*y*_ *connects to the other side of terminal Rt*_*x*,*y*_ *by using the T-Links*. *The T-Links can be T-L*^*NE*^, *T-L*^*NW*^, *T-L*^*SE*^ *and T-L*^*SW*^.

The three types of M-, T- and CBP-Links for Rt_x, y_ router are shown in Fig 3A–3C.

The assignment algorithm related to M-, T- and CBP-Links for proposed CBP-Torus is given in appendix A.

For example, Fig 2 depicts a 5×5 CBP-Torus with origin at point (0,0). Here, router Rt_2,2_ is connected to its adjacent neighbors Rt_1,2_, Rt_3,2_, Rt_2,3_, and Rt_2,1_ via links at its ports M-L^N^, M-L^S^, M-L^E^, and M-L^W^. Further, Rt_2,2_ is connected via CBP-Links at the ports CBP-L^NW^, CBP-L^NE^, CBP-L^SW^, and CBP-L^SE^ with the routers Rt_1,1_, Rt_1,3_, Rt_3,1_, and Rt_3,3._ Similarly Rt_0,0_ has M-Links and CBP-Links along with two T-Links connected to the other side of terminal routers of Rt_0,4_, Rt_4,0_ with T-L^E^ and T-L^S^.

### Effectiveness of Links in CBP-Torus

**Fig 4:**
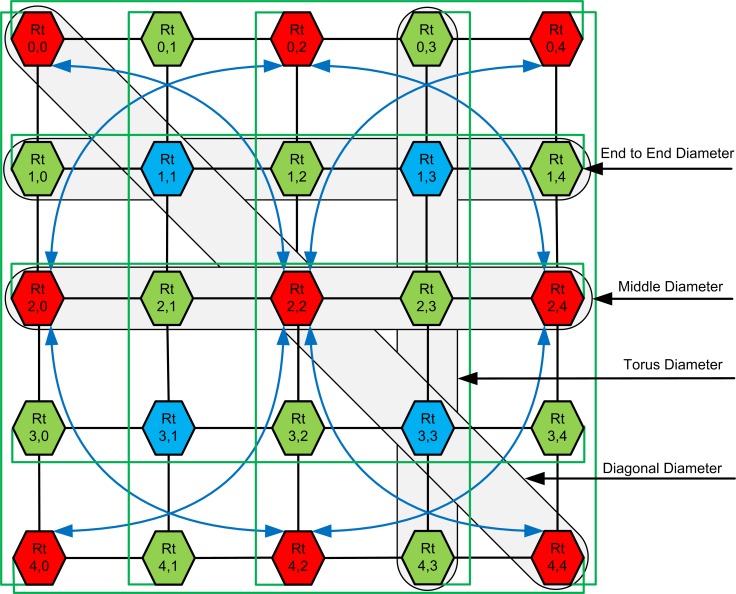
The four type of network diameters for CBP-Torus.

In Fig 4 three different color routers (red, green and blue) are shown with hexagonal-box and interlinking each other with three different colors for links (blue, green and black). The red routers have one, two or four CBP-links (see blue lines) in addition to M-Links (see black lines). The all terminal nodes also have T-Links (see green lines) in the router. The green color router has one and blue router has two hops distance to the nearest CBP-links router nodes. The CBP-links connect the longer distance nodes by over-passing the in-between nodes (like fly over on the roads) of the network. Fig 4 illustrates CBP-Torus network, reducing the network diameter and the distance between nodes.

The CBP-Links connect the corner nodes (Rt_0,0_, Rt_0,4_, Rt_4,0,_ Rt_4,4_ ↔ Rt_2,2_) to a central node in one hop. The middle terminal nodes (Rt_0,2,_ Rt_4,2_ ↔ Rt_2,0_, Rt_2,4_) take also one hop to connect (see blue arrow lines in Fig 4). Similarly corner nodes (Rt_0,0_, Rt_0,4_, Rt_4,0,_ Rt_4,4_) via center node (Rt_2,2_) take two hops to traverse in-between nodes. The middle terminal nodes (Rt_0,2_ ↔ Rt_4,2_) via (Rt_2,0_ or Rt_2,4_) and (Rt_2,0_↔ Rt_2,4_) via (Rt_0,2_ or Rt_4,2_) to connect with each other and take one hop. The adjacent green router nodes will take one more and blue router nodes will take two more hops using M-Links from the above router nodes in CBP-Torus network. The T-Links in network connects the other side of terminals like a loop (see green lines in Fig 4). Each T-Link in CPB-Torus reduces the distance in the same coordinate nodes maximum by half [19]. Further advantages are the connection of CBP-Links and T-Links to the central/terminals of the network (see Fig 4), which provides improved traffic flow and reduced hop count. For example, the hop count between nodes Rt_0,0_ ↔ Rt_4,4_ or nodes Rt_0,4_ ↔ Rt_4,0_ reduces from nine in a 5×5 Mesh network to two hops in the CBP-Torus network.

The gray areas in Fig 4 indicate four types of network diameters for the m×n CBP-Torus, namely the diagonal diameter (*D*_*Di*_), the end to end diameter (*E*_*Di*_), middle diameter (*M*_*Di*_) and the Torus diameter (*T*_*Di*_). These diameters can be computed for symmetric CBP-Torus with dimension n×n following Eqs (1–5):

$$D_{Di}=n-(\left\lfloor \frac{n}{2}\right\rfloor +1)$$ (1)

$$E_{Di}=n-\left\lfloor \frac{n}{2}-1\right\rfloor$$ (2)

$$M_{Di}=n-\left\lfloor \frac{n}{2}\right\rfloor \;(n=\{3,7,11,15,\ldots \}$$ (3)

$$M_{Di}=n-\left\lceil \frac{n}{2}\right\rceil \;(n=\{5,9,13,17,\ldots \}$$ (4)

$$T_{Di}=\left\lfloor \frac{n}{2}\right\rfloor$$ (5)

**Fig 5:**
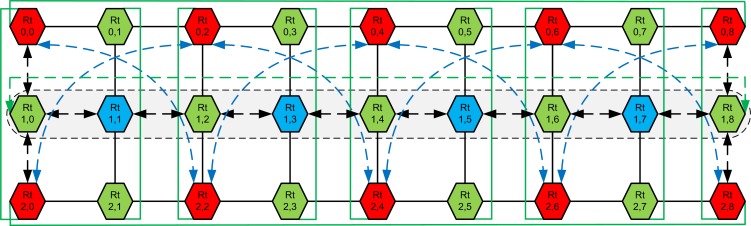
The 3×9 CBP-Torus network.

As shows 3×3 network in Fig 1 (h) is the basic scalability building block of CBP-Torus. A CBP-Torus architecture can be extended to odd (5×5) depicted in Fig 3 or or higher number of nodes in the network as shown in Fig 5 and also can be extended to any size of odd/or even network. As the proposed CBP-Torus scale-up, the CBP-Links and T-Links become more effective in reducing the distance between nodes in the network. The 3×9 CBP-Torus scale is shown in Fig 5. The Rt_0,0_ ↔ Rt_0,4_ and Rt_2,0_ ↔ Rt_2,4_ (see blue dotted arrow in Fig 5) reduces the hop count to two as opposed to four in Mesh, Torus, D-Mesh and D-Torus networks. Similarly Rt_0,0_ ↔ Rt_0,6_ and Rt_2,0_ ↔ Rt_2,6_ will take three hops by using the CBP-links and adjust green router nodes take one more hop to traverse the packets.

Moreover, in the gray area Fig 5 indicates the path between nodes Rt_1,0_ and Rt_1,6_ in a 3×9 network, which would have a hop count of six in a Mesh and other selected network. In contrast, in the proposed CBP-Torus the hop count reduces to five (see double arrow lines in Fig 5). For networks with larger amount of nodes, the gain due to CBP- and T-Links increases considerably. For example, in the 3×9 network depicted in Fig 5, the hop counts between extreme nodes Rt_1, 0_ and Rt_1,8_ reduce from 8 for a common Mesh to 6 in case of the proposed CBP-Torus by using CBP-Links. The T-links reduce this by one hop (see T-Link with green dotted lines in Fig 5).

The existence of alternative paths between two nodes increases the tolerance of the network against failing links and routers. Consequently, the proposed CBP-Torus having T-, CBP- and M-links in a network give more robust than the classic Mesh, Torus, C^2^-Mesh C^2^-Torus and CBP-Mesh topologies.

## Characteristics of CBP-Torus Architecture

The addition of links impacts the topology characteristics which include network diameter, bisection width, degree of routers, number of links and path diversity and average distance of network [19]. The selected topologies’ characteristics as follows, whereas symmetric (*n × n*) sizes are assumed.

### Network Diameter

The network diameter is the minimum number of hop counts between farthest terminal node pairs of network [3]. By reducing the network diameter, hop counts between nodes is minimized leading to the reduced overall latency of the network. Each dimension of mesh can be made symmetrical by taking an equal number of rows and columns (*n × n*). Therefore, the mesh network diameter would be (*2n-2*) [20]. The reduced diameter of CBP-Mesh is shown in Eq (6) realized with CBP-Links in the network. Network diameter of Torus by terminal connections is shown in Eq (7). The average network diameter of CBP-Torus can be the average network diameters of both the Torus and CBP-Mesh topologies. The proposed CBP-Torus average network diameter can be represented by Eq (8).

$${Di}_{CBP\_mesh}=n-1$$ (6)

$${Di}_{Torus\_terminal}=\frac{n}{2}$$ (7)

$${Di}_{Avg\_CBP\_Torus}=\frac{3n-2}{4}$$ (8)

### Bisection Width

Bisection width is the smallest width in the network, which divides (*n × n*) Mesh nodes of network into equal sets of nodes [18]. The bisection width of Mesh network is specified by (*n*) [19]. Adding links in network architecture design increase the value of (*n*), which gives better throughput and traffic flow in the network [23]. To divide a CPB-Mesh network with (*n × n*) nodes into two equal sets of nodes, is given as (2*n*) when topology is even and (*2n + 1*) when it is odd. Similarly, for CBP-Torus bisection width is (*3n*) for even and (*3n + 2*) for odd topology.

### Degree of Router

Five degrees are needed for all routers in Torus topology. Mesh, D-Mesh, D-Torus, C^2^-Mesh and CBP-Mesh and proposed CBP-Torus topologies consist of varying degrees of links for routers such as three, four, five, six, seven and nine, depending upon the nature of the network, detail including local port is given in Table 1.

**Table 1:** Router with different port numbers required in 5×5 network for selected topologies.

Topology	3-Port	4-Port	5-Port	6-Port	7-Port	9-Port	No. of Links
Mesh	4	12	9	0	0	0	40
Torus	0	0	25	0	0	0	50
D-Mesh	0	4	0	12	0	9	56
D-Torus	0	0	0	4	12	9	66
C^2-^Mesh	0	16	8	0	0	1	44
C^2^-Torus	0	0	20	4	0	1	54
CBP-Mesh	0	8	8	4	0	1	48
CBP-Torus	0	0	16	4	4	1	58

### Number of links

The number of links required to construct (*n × n*) Mesh network is (*2n*^*2*^*-2n*) whereas (*2n*^*2*^) links are required for a Torus network [23]. It can be interpreted from Fig 3 that CBP-Torus architecture increases the router degree in some routers due to increase in number of links, however improvement in bisectional width gives better control over traffic flow and enhancement of throughput in the network shown in Table 1.

### Path Diversity

CBP-Torus topology shows the existence of multiple paths between all node pairs of the network in Fig 3. Therefore, each node pair has more than one path for traversing packets from source to destination which increases the fault tolerance capability of the network. In proposing CBP-Torus, three types of path are available to route the data packets in the network. Fig 3 depicts the Mesh, Torus and CBP-Links by black, blue and green lines respectively.

### Average Distance

The average distance of ‘*N*’ node network (*D*_*avg*_) given in Eq (9) is calculated by the minimum hop count from source-nodes to destination-nodes [24–25]. D_SP_ is the shortest path from the source node (*Rti*) to the destination node (*Rtj*) specified in units of hops.

$$D_{avg}=\frac{1}{N^{2}}\sum_{i,j\in N}D_{sp}[\left({Rt}_{i}),({Rt}_{j}\right)]$$ (9)

The computation results in Table 2 showed that CBP-Torus traverses less average distance in different scale size networks compared to other selected topologies.

**Table 2:** Average distance for selected topologies.

Network Size	Mesh	Torus	D-Mesh	D-Torus	C^2^-Mesh	C^2^-Torus	CPB-Mesh	CPB-Torus
3x3	1.89	1.44	1.39	1.33	1.67	1.39	1.54	1.31
5x5	3.32	2.47	2.49	2.25	2.51	2.31	2.33	2.11

Table 3 summarizes the network characteristic for the selected topologies.

**Table 3:** Network characteristic of selected topologies.

Characteristics	Mesh	Torus	D-Mesh	D-Torus	C^2^-Mesh	C^2^-Torus	CPB-Mesh	CPB-Torus
No. of Nodes	n^2^	n^2^	n^2^	n^2^	n^2^	n^2^	n^2^	n^2^
Diameter	2n-2	n-1	n-1	n-1	n-1	n-1	n-1	(3n-2)/4
Bisection	n	2n	3n-2	4n-2	n+2	2n +2	2(n+1)	3n+2
Number of Links	2n^2^-2n	2n^2^	4n^2^-6n+2	4n^2^-4n+2	2n^2^-2n+4	2n^2^+4	2n^2^-2n+2⌊*n*/2⌋^2^	2n^2^+2⌊*n*/2⌋^2^
Router Degree	3 to 5	5	4 to 9	5 to 9	4 to 9	5 to 9	4 to 9	5 to 9
Path Diversity	Yes	Yes	Yes	Yes	Yes	Yes	Yes	Yes

## Performance Vs Cost Comparison

Performance for NoC can be measured in terms of average latency, throughput, power and energy of the network [26–30]. Different NoC networks need a different number of routers with varying degree of ports to link routers and nodes in the networks. To analyze the behavior and effectiveness of the proposed topology, a comparison is presented as performance versus cost of the network. The selected topologies are the classic Mesh and Torus, some of CBP-Torus predecessor C^2^-Torus and CBP-Mesh and its competitor D-Torus.

### Simulation experiments

The NoCTweak [31] simulator was used to implement the classic Mesh, Torus, C^2^-Torus, CBP-Mesh, D-Torus and proposed CBP-Torus and analysis of all the NoC topologies. The simulator is an open source and cycle-level accurate tool written in SystemC [31]. NoCTweak was selected for simulation due to the availability of large sets of workloads. The synthetic traffic model and some real embedded system application workloads are considered for simulations. The simulator provides results in terms of average network latency, throughput and total network power and energy. The simulator configurations used are wormhole 3-stage pipeline routers with ten-flit buffers, round-robin arbiters and 1000-μm links, 65 nm CMOS, 1.0 V operating voltage and 1.0 GHz frequency. Each simulation runs for 100,000 cycles with 20,000 cycles of warm-up cycle time. The existing source routing algorithm to compute the shortest path and NMAP algorithm to map embedded application on the processing cores of network are used [31]. The uniform random traffic traces and packet length of ten flits at a flit injection rate of 0.30 flits/cycle/node over the five different network sizes 3×3 to 7×7 and 9×9 are used for simulation and analysis of selected topologies vs proposed on-chip architectures.

**Fig 6:**
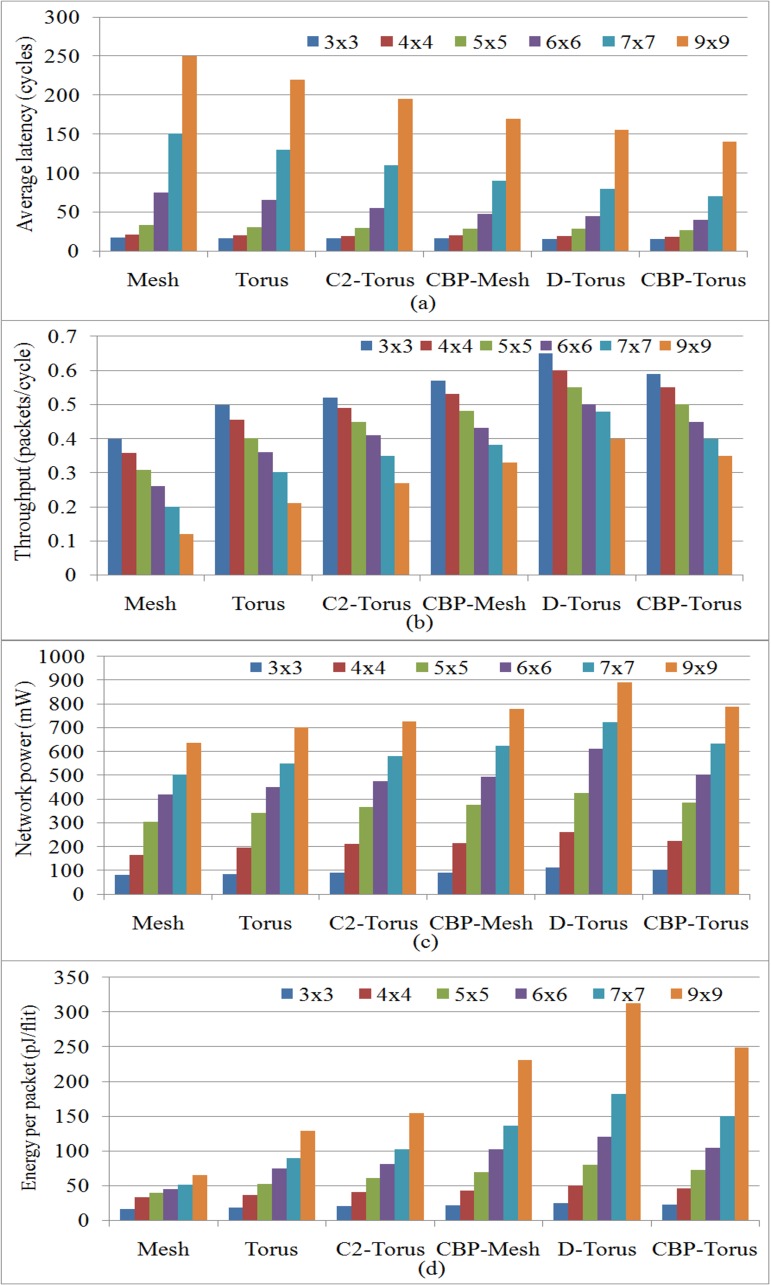
The compassion of performance and power using the uniform traffic for the proposed and selected topologies. a) Average network latency, b) Average network Throughput, c) Total network power, d) Energy per data transferred packets.

The results of latency and throughput are depicted in Fig 6A and 6B) showing that the Mesh topology is worst case for latency and throughput among other topologies. But Mesh has also taken low cost in terms of total network power and energy due to simple network design as shown in Fig 6C and 6D) Whereas CBP-Torus topology is the best candidate among Mesh, Torus, C^2^-Torus, CBP-Mesh and D-Torus as it takes less average network latency in different scale networks. Fig 6(B) also indicates that CBP-Torus gives higher throughput in the different scale networks and is the second best among other selected topologies except D-Torus. D-Torus gives the highest throughput against other networks. As all nine degree routers are required for inner nodes and highest number of links (see in Table 3) to implement D-Torus network topology, it increases the cost of power and energy as compared to other topologies (see Fig 6C and 6D). The proposed CBP-Torus topology uses different degree routers and less number of links as compared to D-Torus to connect the network (sees in Table 1). Hence, CBP-Torus takes less power consumption and energy utilization as compared to D-Torus (see Fig 6C and 6D). The addition of links and increased ports of routers in CBP-Torus increases the cost of power which is evident from Fig 6(C).

### Embedded Applications

Besides the synthetic traffic, the NoCTweak simulator provides several real time embedded application traces. A NMAP algorithm is adopted to convert the task-graph for placement of tasks of the application on the cores of the NoC. Table 4 shows some embedded applications selected for comparisons of topologies.

**Table 4:** Some selected embedded applications.

Applications	Embedded applications required task
mpeg4	MPEG4 decoder with 12 cores
Wifirx	WiFi baseband receiver with 25 cores
Vopd	Video object plane and decoder with 16 cores
Vce	Video conference encoder with 25 cores
mms	Multimedia system with 25 cores

**Fig 7:**
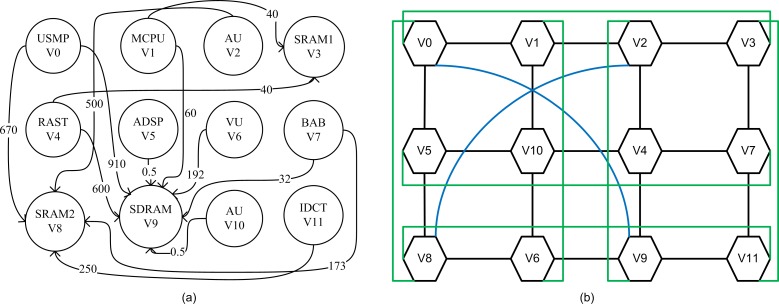
a) Task-graph of MPEG-4 decoder application b) Implementation MPEG-4 decoder application on CBP-Torus.

The complete task graph of one of the chosen applications i.e; MPEG-4 decoder having 12 cores V0 to V11 is shown in Fig 7(A). The bandwidth required for communication between different tasks is depicted with arrow lines in Fig 7(A).

The mapping of MPEG4 decoder application on CBP-Torus using NMAP algorithm is shown in Fig 7(B). The addition of M-, T-, and CBP-links in CBP-Torus network minimizes the paths between nodes of V_0_ → V_9_ and V_2_ → V_8_ connected directly with the CBP-Links (see the blue lines in Fig 7B) The V_0_, V_11_→ V_8_ also directly connected with the T-links in a network (see green lines in Fig 7B).

**Fig 8:**
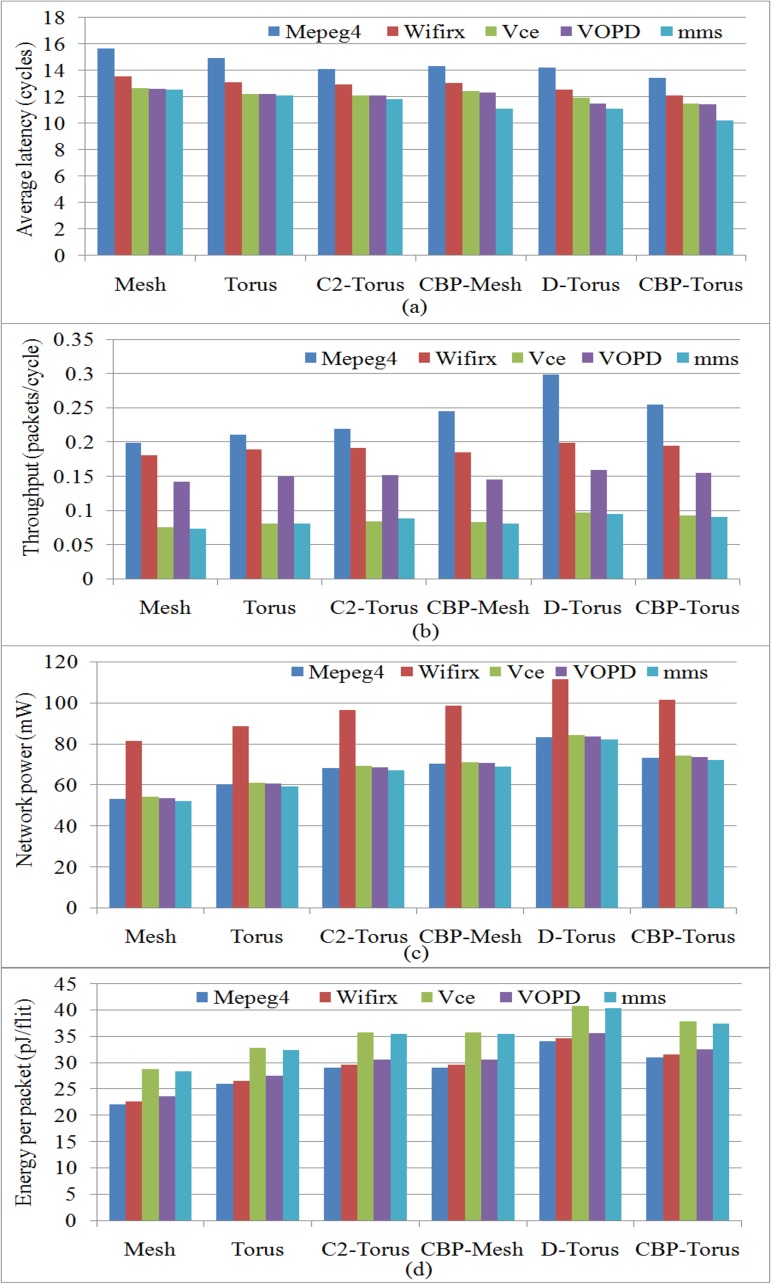
The compassion of performance and power using different embedded application traffic workloads for the proposed and selected topologies. a) Average network latency, b) Average network throughput, c) Total network power, d) Energy per data transferred packets.

The comparison of average network latency, throughput, total network power and energy under the workload of five different embedded applications are shown in Fig 8A–8D). The CBP-Torus takes less average latency cycles as compared to Mesh, Torus, C^2^-Torus, CBP-Mesh and D-Torus by 14.2%, 11.5%, 7.4%, 6.4% and 5.1% respectively under the embedded traffic of MPEG-4 decoder application. CBP-Torus also produces high throughput as opposed to Mesh, Torus, C^2^-Torus and CBP-Mesh by 28%, 20%, 16%, and 8% except from D-Torus which is less than 15%. The proposed architecture takes more network power for MPEG4 application than Mesh, Torus, C^2^-Torus, CBP-Mesh by 37.7%, 21.2%, 7.5%, 4.2% but 13.6% less than D-Torus. It is evident From Fig 8A–8D that under the traffic of all the selected applications, CBP-Torus takes less average network latency cycles than Mesh, Torus, C^2^-Torus, CBP-Mesh and D-Torus topologies.

## Results and Discussion

To show the scalability of the proposed network, different sizes of networks such as 3×3 to 9×9 were used for simulation and analysis of selected topologies. The synthetic traffic trace is applied as workload to all the networks in order to get a fair comparison shown in Fig 6A–6D in terms of average network latency, throughput, total network power and energy of data packets transferred. In order to achieve good performance in NoC Mesh network, some authors modified the design and presented D-Torus network to increase the performance of Mesh and Torus topologies. However, they achieved lower latency at the high cost of power consumption and energy utilization of the network. C^2^-Torus topology showed improved performance with increase in cost, but it is not comparable with D-Torus like topologies in terms of performance. CBP-Torus provides a better trade off with low latency among all others and lower power consumption against D-Torus network. CBP-Torus gives less average latency with better throughput among its predecessor and competitor topologies under both the synthetic as well as embedded application as shown in Fig 6A–6D and Fig 8A–8D. The CBP-Torus proved to be more effective in reducing the network diameter because terminal node pair links are connected with CBP-Links which provides the best connectivity in the network. The addition of such features reduces network diameter and number of hops between nodes in the network.

## Conclusion

Intelligent placement extra links in 2D Mesh architecture for interconnecting the nodes of the network can play an important role in achieving high performance with low cost. Proposed CBP-Torus is the modified design of 2D Mesh architecture that can achieve goals of high performance and low power. The Proposed design integrated the features of CBP-Mesh and Torus topologies to reduce the latency in the network. The introduction of M-, T- and CBP-links in CBP-Torus architecture design achieves the goals of reducing the network diameter, minimizing the average number of hops in the network and providing multi-paths for the adoption of 2D based adaptive routing algorithms. CBP-Torus also provides fault tolerance due to the presence of additional paths between node pairs. Comparison of performance versus cost for proposed CBP-Torus compared to its predecessor and competitor topologies is analyzed. The results show that CBP-Torus takes lowest average latency with good throughput among its predecessor and competitor topologies under both kinds of traffic traces i.e; synthetic and embedded applications. CBP-Torus gives better performance among other selected meshes with a slight increase of cost from its predecessor and low cost against its competitor topologies. The scalable routing algorithm for CBP-Torus will be proposed in the future work.

## Appendix A

Link assignment algorithm for a CBP-Torus network with size m×n. The current router node is r_t_(x,y) and the connecting links to neighbouring routers with M-Links are M-l^N^, M-l^S^, M-l^E^ M-l^W^, T-Links are T-l^N^, T-l^S^, T-l^E^, T-l^W^ and CBP-Links are C-l^NE^, C-l^SE^, C-l^NW^, C-l^SW^.

BEGIN

{

for i = 0:i< = m-1

for j = 0:j< = n-1

{x = i, y = j;

// assigns links to the four terminal corners nodes

if (r_t_(x,y) = = (0,0)) // then assign links

r_t_(x,y)← M-l_E_ with r_t_(x,y+1),

r_t_(x,y) ← M-l_S_ with r_t_(x+1,y),

r_t_(x,y) ← CBP-l_SE_ with r_t_(x+2,y+2),

r_t_(x,y) ← T-l_N_ with r_t_(0,n-1),

r_t_(x,y) ← T-l_w_ with r_t_(m-1,0);

else if (r_t_(x,y) = = (0,n-1)) // then assign links

r_t_(x,y) ← M-l_W_ with r_t_(x,y-1),

r_t_(x,y) ← M-l_S_ with r_t_(x+1,y),

r_t_(x,y) ← CBP-l_SW_ with r_t_(x-2,y-2),

r_t_(x,y) ← T-l_N_ with r_t_(m-1,n-1),

r_t_(x,y) ← T-l_E_ with r_t_(0,0);

else if (r_t_(x,y) = = (m-1,0)) // then assign links

r_t_(x,y) ← M-l_N_ with r_t_(x-1,y),

r_t_(x,y) ← M-l_E_ with r_t_(x,y+1),

r_t_(x,y) ← CBP-l_NE_ with r_t_(x-2,y+2);

r_t_(x,y) ← T-l_W_ with r_t_(m-1,n-1),

r_t_(x,y) ← T-l_S_ with r_t_(0,0);

else if (r_t_(x,y) = = (m-1,n-1)) // then assign links

r_t_(x,y) ← M-l_N_ with r_t_(x-1,y),

r_t_(x,y) ← M-l_W_ with r_t_(x,y-1),

r_t_(x,y) ← CBP-l_NW_ with r_t_(x-2,y-2),

r_t_(x,y) ← T-l_S_ with r_t_(0,n-1),

r_t_(x,y) ← T-l_E_ with r_t_(m-1,0);

// assigns links top middle terminal nodes

else if (r_t_(x,y) = = (0,(|y/2| = = 0))) // then assign links

r_t_(x,y) ← M-l_E_ with r_t_(x,y+1),

r_t_(x,y) ← M-l_W_ with r_t_(x,y-1),

r_t_(x,y) ← M-l_S_ with r_t_(x+1,y),

r_t_(x,y) ← CBP-l_SE_ with r_t_(x+2,y+2),

r_t_(x,y) ← CBP-l_SW_ with r_t_(x-2,y-2),

r_t_(x,y) ← T-l_N_ with r_t_ (m-1,y);

else if (r_t_(x,y) = = (0,(|y/2| = = 1))) // then assign links

r_t_(x,y) ← M-l_E_ with r_t_(x,y+1),

r_t_(x,y) ← M-l_W_ with r_t_(x,y-1),

r_t_(x,y) ← M-l_S_ with r_t_(x+1,y),

r_t_(x,y) ← T-l_N_ with r_t_ (m-1,y);

// assigns links to West middle terminal nodes

else if (r_t_(x,y) = = ((|x/2| = = 0),0)) // then assign links

r_t_(x,y) ← M-l_E_ with r_t_(x,y+1),

r_t_(x,y) ← M-l_N_ with r_t_(x-1,y),

r_t_(x,y) ← M-l_S_ with r_t_(x+1,y),

r_t_(x,y) ← CBP-l_NE_ with r_t_(x-2,y+2),

r_t_(x,y) ← CBP-l_SE_ with r_t_(x+2,y+2),

r_t_ (x,y) ← T-l_W_ with r_t_ (x,n-1);

else if (r_t_(x,y) = = (0,(|y/2| = = 1))) // then assign links

r_t_(x,y) ← l_E_ with r_t_(x,y+1),

r_t_(x,y) ← l_N_ with r_t_(x-1,y),

r_t_(x,y) ← l_S_ with r_t_(x+1,y),

r_t_ (x,y) ← T-l_W_ with r_t_ (x,n-1);

// assigns links to East middle terminal nodes

else If (r_t_(x,y) = = ((|x/2| = = 0), (n-1))) //then assign links

r_t_(x,y) ← M-l_W_ with r_t_(x,y-1),

r_t_(x,y) ←M-l_N_ with r_t_(x-1,y),

r_t_(x,y) ← M-l_S_ with r_t_(x+1,y),

r_t_(x,y) ←CBP-l_NW_ with r_t_(x-2,y-2),

r_t_(x,y) ← CBP-l_SW_ with r_t_(x-2,y+2),

r_t_(x,y) ←T-l_E_ with r_t_ (x,0);

else If (r_t_(x,y) = = ((|x/2| = = 1), (n-1))) //then assign links

r_t_(x,y) ← M-l_E_ with r_t_(x,y-1),

r_t_(x,y) ← M-l_N_ with r_t_(x-1,y),

r_t_(x,y) ← M-l_S_ with r_t_(x+1,y),

r_t_(x,y) ←T-l_E_ with r_t_ (x,0);

// assigns links to bottom middle terminal nodes

else If (r_t_(x,y) = = ((m-1), (|x/2| = = 0)))// then assign links

r_t_(x,y) ← M-l_N_ with r_t_(x-1,y),

r_t_(x,y) ← M-l_W_ with r_t_ (x,y-1),

r_t_(x,y) ← M-l_E_ with r_t_ (x+1,y),

r_t_(x,y) ← CBP-l_NW_ with r_t_(x-2,y-2),

r_t_(x,y) ← CBP-l_NE_ with r_t_(x-2,y+2),

r_t_ (x,y) ← T-l_S_ with r_t_ (0,y);

else If (r_t_(x,y) = = ((m-1), (|x/2| = = 1))) then assign links

r_t_(x,y) ← M-l_N_ with r_t_(x-1,y),

r_t_(x,y) ← M-l_E_ with r_t_(x+1,y),

r_t_(x,y) ← M-l_W_ with r_t_(x-1,y),

r_t_ (x,y) ← T-l_S_ with r_t_ (0,y);

// assigns links to middle nodes

else If (r_t_(x,y) = = ((|x/2| = = 0),(|y/2| = = 0))) //then assign links

r_t_(x,y) ← M-l_N_ with r_t_(x-1,y),

r_t_(x,y) ← M-l_S_ with r_t_(x+1,y),

r_t_(x,y) ← M-l_E_ with r_t_(x,y+1),

r_t_(x,y) ← M-l_W_ with r_t_(x,y-1),

r_t_(x,y) ← CBP-l_NW_ with r_t_(x-2,y-2),

r_t_(x,y) ← CBP-l_NE_ with r_t_(x-2,y+2),

r_t_(x,y) ← CBP-l_SW_ with r_t_(x+2,y-2),

r_t_(x,y) ← CBP-l_SE_ with r_t_(x+2,y+2);

else

r_t_(x,y) ← M-l_N_ with r_t_(x-1,y),

r_t_(x,y) ← M-l_S_ with r_t_(x+1,y),

r_t_(x,y) ← M-l_E_ with r_t_(x,y+1),

r_t_(x,y) ← M-l_W_ with r_t_(x,y-1);

}

}

END
